# Treelength Optimization for Phylogeny Estimation

**DOI:** 10.1371/journal.pone.0033104

**Published:** 2012-03-19

**Authors:** Kevin Liu, Tandy Warnow

**Affiliations:** Department of Computer Science, University of Texas at Austin, Austin, Texas, United States of America; University of Wyoming, United States of America

## Abstract

The standard approach to phylogeny estimation uses two phases, in which the first phase produces an alignment on a set of homologous sequences, and the second phase estimates a tree on the multiple sequence alignment. POY, a method which seeks a tree/alignment pair minimizing the total treelength, is the most widely used alternative to this two-phase approach. The topological accuracy of trees computed under treelength optimization is, however, controversial. In particular, one study showed that treelength optimization using simple gap penalties produced poor trees and alignments, and suggested the possibility that if POY were used with an affine gap penalty, it might be able to be competitive with the best two-phase methods. In this paper we report on a study addressing this possibility. We present a new heuristic for treelength, called BeeTLe (Better Treelength), that is guaranteed to produce trees at least as short as POY. We then use this heuristic to analyze a large number of simulated and biological datasets, and compare the resultant trees and alignments to those produced using POY and also maximum likelihood (ML) and maximum parsimony (MP) trees computed on a number of alignments. In general, we find that trees produced by BeeTLe are shorter and more topologically accurate than POY trees, but that neither POY nor BeeTLe produces trees as topologically accurate as ML trees produced on standard alignments. These findings, taken as a whole, suggest that treelength optimization is *not* as good an approach to phylogenetic tree estimation as maximum likelihood based upon good alignment methods.

## Introduction

Most phylogenies are estimated in two steps: first, a multiple sequence alignment is produced, and then a tree is estimated on the multiple alignment. Such “two-phase” methods are reasonably fast and accurate for small enough datasets, but can have unacceptably high error for large datasets that evolve with many indels and substitutions [1], [2].

Methods that co-estimate trees and alignments have also been developed, including [3]–[19]. Some of these methods are likelihood-based methods that are based upon stochastic models of evolution that include indels as well as substitutions [3]–[12]. These likelihood-based methods are computationally very intensive and cannot be used on datasets with more than, perhaps, 200 sequences (BAliPhy [3] is the most computationally scalable of these methods, but it also has not been used on datasets bigger than this). Other co-estimation methods include [13]–[19]; these are generally much faster (and more scalable) than the statistically-based methods. Of these methods, POY [18], [19] is the most commonly used.

POY is a method that tries to optimize a variant of maximum parsimony [20] in which indels contribute to the cost of the tree. Thus, the input to POY is a set of unaligned sequences and an edit distance function, with the edit distance function defined by a cost for every substitution and a gap penalty (defined by a gap open and gap extend cost). The objective criterion in POY is to minimize the total length, as defined by the sum of the edit distances on the edges of the tree. This is the NP-hard treelength problem, originally posed by Sankoff and Cedergren [21]. When indels are forbidden (by setting the gap open cost to infinity), treelength optimization is identical to maximum parsimony [20]; hence, treelength is a generalization of the maximum parsimony criterion. The output of POY is a tree 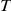 with leaves bijectively labelled by the input sequences and ancestral sequences at every node in the tree, and thus also an alignment on the sequences defined by the tree, ancestral sequences, and edit distance function (i.e., the edit distance function implies an optimal pairwise alignment for every edge between the sequences labelling the endpoints of the edge, and the transitive closure of that set of pairwise alignments is the output multiple sequence alignment).

The use of POY (and of its underlying optimization criterion, treelength) is a matter of controversy in phylogenetics [22]–[28]. For example, in 2007, Ogden and Rosenberg [24] showed that various standard ways of running POY produced trees and alignments that were much less accurate than those computed by maximum parsimony analyses of ClustalW alignments (denoted by MP(ClustalW)). A later study by Lehtonen [29] showed that using more intensive heuristics to optimize simple gap penalty treatments with the newer version of POY produced trees with comparable topological accuracy to MP(ClustalW) [30], even though the alignments were less accurate than ClustalW alignments.

Liu et al. [31] revisited the question by focusing on the specific gap penalty used in POY. They selected a treelength criterion they termed “Affine”, where each gap of 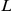 nucleotides had cost 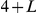, each transition had cost 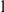, and each transversion had cost 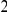. They observed that POY did not optimize the Affine treelength very well, and developed a new method, called POY*, that uses Probtree [32] as the starting tree and then runs POY under the Affine criterion. Liu et al. [31] showed that trees produced using POY* were at least as accurate as trees estimated using maximum likelihood on alignments produced using many popular alignment methods, and concluded that optimizing trees using treelength optimization might produce highly accurate trees under the Affine treelength criterion provided better heuristics for treelength were used.

However, the inference made by Liu et al. [31] that Affine treelength might be a good optimization criterion was based upon the observation that POY* produced highly accurate trees. Since their study showed that POY was not very effective at finding trees that optimized the Affine treelength criterion, it is possible that the topologically accurate trees produced by POY* resulted from the fact that the starting tree was highly accurate, and that the search heuristic used by POY did not move far away from its starting tree.

In this paper we evaluate whether the conclusion by Liu et al. that optimizing Affine treelength is competitive in topological accuracy with many two-phase methods is sustained when a more careful search for short trees is used. To enable this study, we developed a very simple heuristic, BeeTLe (Better TreeLength), that has the following structure: BeeTLe runs a collection of methods, including POY, to produce a set of trees on a given input set of unaligned sequences, uses POY to compute the treelength of each tree, and then returns the tree that had the shortest treelength. Thus, BeeTLe is guaranteed to find trees at least as short as those found using POY, and thus enables us to evaluate the impact of using treelength to find trees.

We report on a study comparing BeeTLe used with three treelength criteria (Affine and two treelength criteria that are based upon simple gap penalty treatments) to POY, two-phase methods, and SATé, a method for co-estimating alignments and trees. We explore performance on simulated and biological datasets, each having at least 100 sequences. We show that BeeTLe produces shorter trees than POY, thus confirming the value in using BeeTLe to optimize treelength instead of POY. We also show that optimizing treelength for all three ways we explored were competitive with maximum parsimony analyses but not with maximum likelihood analyses on almost all alignment methods. Furthermore, alignments produced using treelength optimization are not as accurate as standard alignments.

Thus, for the datasets we explored, treelength optimization was not competitive with the best two-phase methods (maximum likelihood on the leading alignments) with respect to the accuracy of alignments and trees.

### Basics

The treelength problem was originally proposed by Sankoff and Cedergren [21], and can be generalized as follows:

#### Definition 1 The Generalized Sankoff Problem (GSP)

*The input is a set* 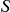 *of unaligned sequences and a function* 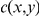 *for the edit cost between two sequences* 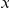 *and* 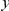 *. The output is a tree* 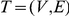 *with leaves labeled by* 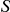 *and internal nodes labeled with additional sequences such that the treelength* 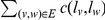 *is minimized, where* 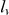 *is the sequence labeling vertex* 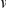.

Thus, GSP is defined by an edit distance function, and this function depends upon how gaps are penalized. GSP is NP-hard, since the case in which the edit distance function forbids gaps (by setting the cost for a gap to be infinite) is the NP-hard Maximum Parsimony (MP) problem [20]. However, the GSP problem is even NP-hard when the tree 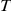 is known (so that the objective is to find the best sequences at the internal nodes of 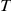 so as to produce the shortest total treelength [33], under a simple gap penalty). Thus the treelength problem is *harder* than the maximum parsimony problem, since even the fixed-tree problem is NP-hard.

Although several algorithms have been developed for the GSP problem (both for the fixed tree and general case), POY is the standard method used to produce trees from unaligned sequences via treelength optimization. POY takes as input a set of unaligned sequences and an edit distance function 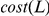 for the cost of a gap of length 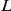, given by 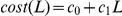. When 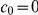 the gap cost is said to be “simple”, and when 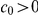 the gap cost is said to be “affine”.

We use three different treelength criteria in this paper, Simple-1, Simple-2, and Affine, as follows. Simple-1 sets the cost of every indel and substitution to 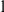. Simple-2 is a treelength criterion studied by Ogden and Rosenberg [24], which they found to produce more accurate trees than any other treelength criterion they considered, and which assigns cost 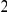 to indels and transversions and cost 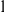 to transitions. Finally, the Affine treelength criterion studied in Liu et al. [31], which produced more accurate trees than Simple-1 or Simple-2, sets the cost of a gap of length 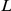 to 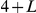.

## Results

**Figure 1:**
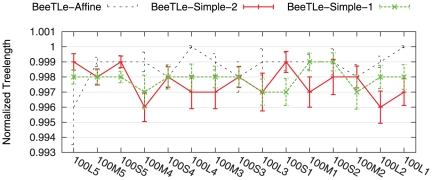
Normalized treelength scores for BeeTLe under different treelength criteria on the 100-taxon model conditions. Treelength scores for BeeTLe under a particular treelength criterion are normalized by POY's score under the same criterion; thus, scores below 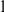 indicate that BeeTLe finds a shorter tree than POY. Averages and standard error bars are shown; 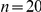 for each reported value.

**Figure 2:**
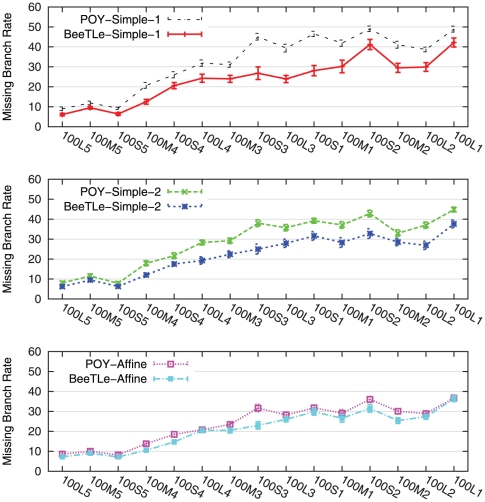
Missing branch rates of POY and BeeTLe under different treelength criteria on the 100-taxon model conditions. Averages and standard error bars are shown; 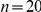 for each reported value.

**Figure 3:**
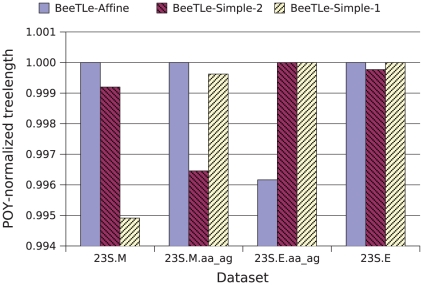
Normalized treelength scores for BeeTLe under different treelength criteria on the biological datasets. Treelength scores for BeeTLe under a particular treelength criterion are normalized by POY's score under the same criterion; thus, scores below 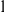 indicate that BeeTLe finds a shorter tree than POY. 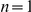 for each reported value.

In our first experiment, we investigated how well POY is able to solve the treelength problems. Figures 1 and 2 compare POY and BeeTLe with respect to treelength and tree error, respectively, for each of the three treelength criteria we consider on simulated data. Comparisons of treelength scores obtained on the biological datasets are provided in Figure 3. Note that for each treelength criterion, BeeTLe generally produces shorter trees. Furthermore, a comparison of topological error rates shows that trees produced by BeeTLe and POY tend to be quite different.

**Figure 4:**
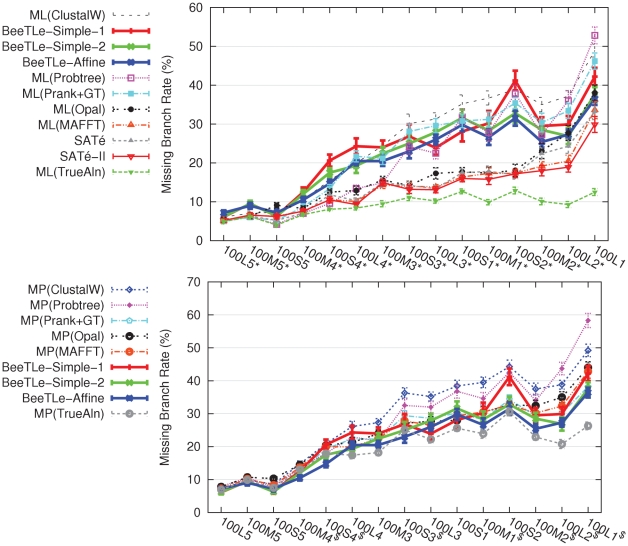
Missing branch rates of different methods on 100-taxon model conditions. We report missing branch rates for BeeTLe-Affine in comparison to ML methods, SATé, and SATé-II (top chart) and in comparison to MP methods (middle chart). On model conditions marked with ‘*’, ML(MAFFT)'s missing branch rate significantly improved upon BeeTLe-Affine's (using one-tailed pairwise t-tests with Benjamini-Hochberg [57] correction for multiple tests, 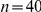 for each test, and 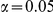). On model conditions marked with ‘$’, BeeTLe-Affine's missing branch rate significantly improved upon MP(MAFFT)'s (using similar statistical tests). Averages and standard error bars are shown; 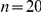 for each reported value.

We then examined the question of whether optimizing treelength can return trees that are competitive with the better two-phase methods with respect to topological accuracy. Our first experiment compared the treelengths achievable for the model (true) and SATé trees (computed using its first version, SATé-I), by letting POY optimize ancestral sequences on these tree topologies. As shown in Table S4, for all three treelength criteria, the model tree had treelengths that were much larger than the shortest treelengths found by POY, and the treelengths for the SATé-I trees were also larger than those found by POY. These observations suggest that optimizing treelength is unlikely to yield highly accurate trees, since the model tree had such poor treelengths compared to those found by POY. However, because BeeTLe is more effective than POY at optimizing treelength, we use BeeTLe to estimate trees and alignments under the three treelength criteria to provide a more critical evaluation of this hypothesis. We compared trees computed using BeeTLe (under each of the three treelength criteria, Simple-1, Simple-2, and Affine) to trees computed using SATé and two-phase methods (maximum likelihood and maximum parsimony on various alignments), in order to determine the impact on topological accuracy of optimizing treelength. We estimated maximum likelihood trees using RAxML [34] and maximum parsimony trees using PAUP* [35], and we used MAFFT [36], Opal [37], Prank+GT (Prank [38] with a particular guide tree, as described in [1]), Probtree [32], and ClustalW [30] to produce alignments. We also used SATé-I [1] and SATé-II [14] to co-estimate alignments and trees. Figure 4 and Table 1 show results on simulated datasets with 100 taxa.

**Table 1:** Average missing branch rate (%) on each 100-taxon model condition.

	100-taxon model condition															Total	Max
Method	L5	M5	S5	M4	S4	L4	M3	S3	L3	S1	M1	S2	M2	L2	L1	Average	Std Err
ML(TrueAln)	4.9	6.2	4.0	6.7	8.1	8.4	9.5	11.1	10.2	12.7	9.9	12.9	10.1	9.3	12.5	9.1	0.9
SATé-II	5.2	6.6	6.2	7.6	10.5	9.3	14.9	13.2	13.1	16.0	15.8	17.2	18.0	18.9	29.9	13.5	2.1
ML(MAFFT)	5.2	6.5	6.3	7.6	10.5	10.1	14.3	14.0	13.8	16.4	17.3	17.3	19.2	20.5	33.6	14.2	2.2
SATé	5.0	6.3	5.2	7.1	11.8	10.3	14.9	14.2	13.4	17.5	17.8	17.0	22.4	24.3	33.2	14.7	2.1
ML(Opal)	5.4	6.3	9.1	8.3	12.5	12.9	15.6	14.2	17.3	17.7	17.4	18.1	23.1	27.7	38.0	16.2	2.0
MP(TrueAln)	6.9	9.9	7.5	13.1	17.9	17.4	18.2	25.1	22.2	25.6	23.9	30.6	22.9	20.8	26.3	19.2	1.4
BeeTLe-Affine	7.2	9.0	7.2	10.5	14.7	20.5	20.5	23.0	26.0	29.8	26.6	31.5	25.4	27.3	36.4	21.0	2.0
ML(Probtree)	5.3	6.2	4.2	7.0	9.6	13.4	14.9	24.2	22.5	31.8	27.8	37.8	27.3	36.0	52.8	21.4	2.6
BeeTLe-Simple-2	6.2	9.5	6.3	12.0	17.5	19.3	22.4	25.0	27.9	31.6	28.3	32.8	28.5	26.9	37.6	22.1	2.5
ML(Prank+GT)	5.0	6.0	5.2	8.7	13.4	21.7	21.2	28.1	29.6	30.8	31.2	35.4	30.4	33.4	46.2	23.1	2.4
MP(Prank+GT)	6.7	9.3	7.1	12.7	19.3	22.4	22.3	29.4	28.6	30.2	28.2	34.3	29.4	30.2	38.2	23.2	1.8
MP(MAFFT)	7.3	10.2	8.3	13.7	20.0	19.9	22.5	27.8	26.5	29.4	30.5	32.7	30.2	32.3	42.8	23.6	2.0
BeeTLe-Simple-1	6.1	9.5	6.4	12.5	20.6	24.3	24.0	26.8	23.9	28.1	30.2	41.2	29.5	29.9	42.2	23.7	3.2
MP(Opal)	7.8	10.7	10.3	14.5	20.4	21.3	23.9	27.1	27.7	28.0	30.4	32.7	32.4	35.0	44.0	24.4	1.8
ML(ClustalW)	6.1	7.0	7.3	10.6	15.5	20.0	24.4	30.1	31.3	35.2	36.7	38.9	35.0	37.2	48.9	25.6	2.5
MP(Probtree)	7.9	10.5	7.8	13.5	18.0	22.9	21.7	32.5	32.0	36.8	34.6	42.6	34.0	43.7	58.3	27.8	2.2
MP(ClustalW)	7.0	9.9	8.6	14.7	20.9	26.1	27.3	36.3	35.2	38.4	39.5	44.3	37.5	38.8	49.2	28.9	2.0

For conciseness, model condition identifiers are truncated to unique suffixes. “Total Average” is the average across all model conditions. “Max Std Err” is the maximum standard error of any model condition. 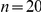 for each reported value; 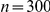 for “Total Average” and “Max Std Err”.

These results show that trees computed using BeeTLe, under any of the three treelength criteria, were generally less topologically accurate than the best alternative methods (i.e., SATé-I, SATé-II, ML(Probtree), and ML(MAFFT)). A comparison of BeeTLe to SATé-II, for example, shows that on model conditions 100L5 and 100M5, BeeTLe trees were almost as accurate as SATé-II trees (1%–3% difference in missing branch rate), and had indistinguishable performance on 100S5. However, these are the slowest evolving models, and not all biological datasets evolve quite this slowly (compare, for example, the empirical statistics of the biological datasets we studied to those of these simulated model conditions). Under the harder model conditions, differences between methods grew, and the methods separated into two distinct classes: the most accurate methods (SATé-I, SATé-II, ML(MAFFT) and ML(Opal)) and the less accurate methods (ML(ClustalW, ML(Probtree), ML(Prank+GT), and all three BeeTLe methods). In particular, ML(MAFFT)'s missing branch rate was significantly better than BeeTLe-Affine's on almost all model conditions (Benjamini-Hochberg-corrected pairwise t-tests with 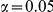, as discussed in Table S3). Furthermore, the difference in tree error between the less and more accurate methods generally increased with the difficulty of the model condition. Thus, trees computed by BeeTLe were clearly much less topologically accurate than those produced by any of the more accurate methods. Within the set of less accurate methods, BeeTLe-Affine did particularly well, and often produced more accurate trees than any of the other less accurate methods. Thus, in general, trees estimated using BeeTLe were far less accurate than ML trees estimated on alignments produced by SATé-I, SATé-II, MAFFT and Opal. A comparison between BeeTLe-Affine, BeeTLe-Simple-1, and BeeTLe-Simple-2 shows that BeeTLe-Affine generally produces more accurate trees than BeeTLe-Simple-1 or BeeTLe-Simple-2.

When we consider trees estimated using Maximum Parsimony (MP), the comparison changed substantially. We see that BeeTLe-Affine was not only competitive with maximum parsimony trees, but that BeeTLe-Affine produced more accurate trees than MP on most alignments for almost all model conditions (the only exception being MP(MAFFT), as shown in Table S3). BeeTLe-Simple-2 was also reasonably accurate, producing more accurate trees than maximum parsimony on ClustalW, ProbTree, Prank+GT, and Opal, for many model conditions.

**Figure 5:**
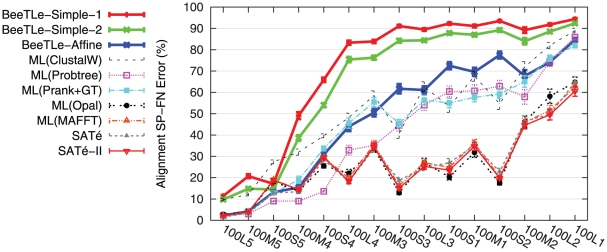
Alignment SP-FN error of different methods on 100-taxon model conditions. Averages and standard error bars are shown; 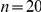 for each reported value.

Although our main concern is topological accuracy, we note that alignments estimated by either POY or BeeTLe, irrespective of treelength optimization criterion, were less accurate than alignments produced by the standard alignments methods, and much less accurate than SATé alignments (Fig. 5).

## Discussion

The most important observation in this study is that for all three treelength criteria we explored, trees and alignments found by methods for optimizing treelength were generally *not* among the most accurate. Thus, maximum likelihood trees produced on almost all alignment methods we studied were more accurate than BeeTLe and POY trees. Interestingly, trees estimated by BeeTLe-Affine and Beetle-Simple-2 *were* often more accurate than maximum parsimony trees, even on very good alignments, showing the potential for treelength optimization to improve phylogenetic estimation. However, treelength optimization did not produce trees of comparable accuracy to trees produced using maximum likelihood, except for very poor alignments.

Our study also showed that BeeTLe is often able to find shorter trees than POY, and ones that are topologically quite different from those found by POY. Thus, inferences about the utility of treelength for phylogeny and alignment estimation can be made more accurately when using BeeTLe than when using POY.

Additional observations can be made that may impact further studies. First, the choice of treelength criterion clearly impacts tree error. In particular, BeeTLe-Affine was consistently at least as accurate as BeeTLe-Simple1 or BeeTLe-Simple2 on the simulated data. A comparison of the gap length distributions between the simulated and biological data (see Figure S1, Figure S2, Table S1, and Table S2) shows that these two types of datasets have very different distributions. The simulated datasets have medians and means that are very close, and have only moderately large maximum gap lengths; by contrast, the biological datasets all have medians equal to 1 (so that more than half of the gaps are single nucleotides) and some very long gaps. In fact, the biological datasets have maximum gap length ranging from 1400 to 3889. Given this combination of features, it is clear that the biological datasets we studied do not have gap lengths drawn from the same distribution as are created by this simulation protocol. More generally, it seems likely that the gap length distribution for these biological datasets may be drawn from a mixture model rather than some simple single parameter model, such as is used in this simulation. Thus, appropriate choices of the gap length penalty may require particular care. More generally, to the extent that optimizing treelength is to be helpful in estimating trees, the choice of treelength criterion will need to be made carefully.

The study we provide has several limitations. First, we explored only three treelength criteria. While these three criteria are “good” choices for treelength (in particular because Simple-2 gave the best results in Ogden and Rosenberg's study [24] and Affine gave even better results in Liu et al.'s subsequent study [31]), they are by no means representative of the full range of treelength criteria. Therefore it remains possible that a better treelength criterion can be developed. However, as noted above, it may be that the use of affine treelengths may be inherently too simplistic (fitting single parameter models rather than mixture models) to produce good results. Also, our method, BeeTLe, is not designed to thoroughly search treespace for short trees. Instead, it is a very simple technique that scores a set of trees (including POY, RAxML(MAFFT), RAxML(ClustalW) and some of the neighbors of these trees) for treelength, and returns the shortest tree. Therefore, it is likely that even shorter trees would be obtained by a more careful search through treespace. As a result it is possible that the shorter and topologically more accurate trees would be obtained by a more careful analysis.

It is worth noting that we only explored two phylogeny estimation methods (i.e., RAxML for ML analysis and PAUP* for MP) and a handful of alignment methods (i.e., MAFFT, SATé, Probtree, Prank+GT, Opal, and ClustalW). It is possible that better alignments could be obtained using other alignment methods and that better trees might be obtained on these alignments using other phylogeny estimation methods. In particular, likelihood-based methods such as MrBayes [39], Phyml [40], GARLI [41], FastTree [42], [43], and Metapiga2 [44] might produce more accurate trees. We also did not explore the performance of BAli-Phy or other co-estimation methods that treat indels informatively, and these also might produce more accurate trees. Thus, it is possible that there are currently available methods that might yield more even more accurate trees than those tested in this study.

We close with some comments about the general problem of estimating trees and alignments from unaligned sequences, and whether co-estimation of trees and alignments is beneficial or detrimental. In other words, although it is important to understand whether POY (or any method based upon treelength optimization) is reliable for estimating highly accurate trees or alignments, the more important question is *which approaches are likely to produce the most accurate trees and alignments?*

The study we presented suggests strongly that treelength optimization is unlikely to produce trees or alignments that are as accurate as maximum likelihood on the leading alignment methods; it also showed that SATé trees and alignments were even more accurate than maximum likelihood trees on leading alignments. Thus, parsimony-style co-estimation (as in POY and BeeTLe) produced trees and alignments that are inferior to the co-estimation approach in SATé. It makes sense, therefore, to discuss SATé 's co-estimation technique.

The technique used by SATé to co-estimate trees and alignments uses iteration combined with divide-and-conquer; each iteration involves the estimation of a new alignment (produced using divide-and-conquer) and then uses RAxML to produce an ML tree on that new alignment. However, the ML model used in estimating the tree is GTR+Gamma, and so indels are treated in the standard way, which is as missing data – rather than treating them as events in a stochastic model that includes indels as well as substitutions. This approach clearly has empirical benefits (as shown in this study and in [1], [14]) over the two-phase methods we studied; however, this is not a statistically rigorous indel treatment method. We hypothesize that methods that treat indels in a statistically rigorous manner are likely to produce more accurate alignments and trees than SATé.

Finally, this entire study (and the previous studies we discussed [24], [29], [31]) are based upon nucleotide sequences, and so even though treelength-based methods, such as POY, can be used on amino-acid sequences, these studies do not directly yield any insight into the problem of estimating trees and alignments from such sequences. However, the same questions can be asked about amino-acid phylogeny and alignment estimation: is it better to estimate the alignment first and then the tree, or to co-estimate them, and which methods give the most accurate alignments and trees? As with nucleotide datasets, most amino-acid phylogenies have been estimated using two-phase methods (i.e., first an alignment is estimated and then a tree based upon that alignment), with the best alignment and phylogeny estimation methods taking the particular properties of amino-acid sequences into account. Therefore, phylogeny estimation methods that are based upon stochastic models of amino-acid evolution are beneficial (see the discussion in [45]), and alignment estimation based upon estimated or known structural features can also provide improvements. Co-estimation methods like BAli-Phy with statistical performance guarantees can also be used on amino-acid sequences, but are very computationally intensive. SATé can also be used on amino-acid sequences, but although it is very fast (and can analyze large datasets), it has no statistical performance guarantees. To date, no performance study has been published that compare any of these co-estimation methods against the leading two-phase methods on amino-acid sequences. Finally, SATCHMO-JS [17] uses HMMs (Hidden Markov Models) to simultaneously construct a tree and alignment from unaligned sequences, and has been shown to be able to produce more accurate alignments than MAFFT [17]. However, SATCHMO-JS can only be used with amino-acid sequences.

Thus, some co-estimation methods have been able to provide improvements in alignment and phylogeny estimation accuracy relative to two-phase methods for both nucleotide and amino-acid analyses, but not all co-estimation methods give the same accuracy. This study has shown that co-estimation methods that are based upon treelength are not in general as accurate as the leading two-phase methods, which use likelihood-based phylogeny estimation to analyze high quality alignments. This and other studies have also shown that two other co-estimation methods, SATé and SATCHMO, can produce highly accurate results, improving upon leading methods, even on large datasets. However, statistical guarantees for co-estimation methods are only provable for those methods that are based upon stochastic models of evolution that include indels and substitutions. Unfortunately, all such methods are computationally intensive, and have not been able to run on large datasets.

It seems likely that statistically rigorous methods for co-estimating alignments and trees may be key to obtaining highly accurate estimations of evolutionary history (of which alignments and trees are both partial hypotheses); however, all current methods of this type are so computationally intensive that they cannot be used in studies that address hundreds of sequences. Future work is needed in order to create reasonably efficient methods with strong statistical guarantees.

## Materials and Methods

We used 300 100-taxon simulated datasets and four biological datasets with up to 278 taxa. On each dataset, in addition to using POY and BeeTLe (for each treelength criterion), we computed phylogenetic trees using SATé (which co-estimates alignments and trees) and various two-phase methods. We computed the tree error for each estimated tree and alignment error for each estimated alignment.

### Simulated datasets

We used 300 simulated 100-taxon datasets provided by Liu et al. [14], which evolved under a range of gap length distributions (S for short, M for medium, and L for long), relative probabilities of indels to substitutions, and overall amount of evolution, using ROSE [46]. We show the empirical statistics for these datasets in Table S1. In all, there are a total of 15 model conditions, and each model condition has 20 datasets. For each simulated dataset 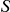 of unaligned sequences we know the model tree and the true alignment. To define the reference tree for each simulated dataset, we follow the methodology of Liu et al. [1], and modify the model tree for the dataset by contracting zero-event branches (that is, branches on which no substitution or indel occurs during the evolutionary process that generates the dataset). This modification is done since reconstruction of zero-event edges is a matter of chance.

### Biological datasets

We used datasets from CRW, the Comparative RNA Website [47]. CRW datasets have reference alignments based upon secondary structure. Based upon these reference alignments, we selected four datasets that present some challenge to alignment estimation due to the large number of indels. Since reference trees were not provided with these datasets, we only used these datasets to investigate the effectiveness of treelength optimization heuristics by comparing treelength scores obtained by different methods. The empirical statistics for these datasets are provided in Table S2.

### Tree and alignment estimation methods

We ran SATé version 7/4/2009 alpha (available from www.cs.utexas.edu/users/phylo/software/sate/) to produce trees and alignments. For the two-phase methods, we computed alignments using several techniques, including MAFFT (using its L-INS-i algorithm) version 6.240 [48]–[50], ClustalW version 2.0.4, Opal version 1.0.2 [37], Probtree (as described in a prior study by Nelesen et al. [32]) using ProbconsRNA version 1.1 [51], and Prank+GT (as described in [1]) using Prank version 080904 [38]. We used RAxML [52], [53] version 7.0.4 to estimate ML trees and a parsimony ratchet analysis using PAUP* [54] version 4.0b10. For our MP analyses, we returned the majority consensus of the best trees that we found. For treelength optimization, we used POY version 4.1.2 and BeeTLe, under the Simple-1, Simple-2, and Affine treelength criteria (defined earlier). The commands used for each method are provided in Methods S1.

We now describe the BeeTLe algorithm. For a given treelength criterion, BeeTLe runs POY under that criterion, and also ML(MAFFT) and ML(ClustalW). BeeTLe then samples the neighborhood around these selected trees, by producing a random perturbation of the selected tree to obtain 20 additional trees. We use random 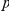-ECR [55] moves to create these perturbations, where a random 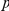-ECR move contracts 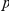 randomly selected edges in a tree, and then randomly refines the resulting unresolved tree to obtain a binary tree. To produce the set of 20 additional trees from a single selected tree, we do the following 20 times: we apply a random 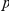-ECR move [55] to the selected tree, with 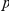 chosen uniformly at random between one and five. This process thus produces 20 additional trees, each of which is one 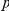-ECR move away from the starting tree.

### Measuring tree error

While the bipartition distance (also known as the Robinson-Foulds distance) is the standard way of measuring tree error, it is inappropriate when either the true or estimated trees are not binary [56], as it is biased in favor of unresolved trees. Since our analyses includes estimated trees that may not be fully resolved, we compare trees using the missing branch rate (also known as the false negative rate), which is the proportion of edges present in the true tree but missing from the estimated tree.

### Statistical significance

We evaluated the statistical significance of the differences in tree error (Table S3) using one-tailed paired t-tests with the Benjamini-Hochberg correction [57].

### Computational resources

The simulations and analyses were performed using a heterogeneous Condor [58] computing cluster at the University of Texas at Austin. This cluster had computers with between 1 and 8 cores running at speeds between 1.86 GHz and 3.16 GHz. All programs were run as 32-bit serial executables on a single dedicated core with dedicated access to at least 512 MB and at most 4 GB of main memory.

## Supporting Information

Figure S1 **Histogram of gap lengths in true alignments from each 100-taxon model condition.** Axes are logarithmically scaled.
(EPS)

Figure S2 **Histogram of gap lengths in reference alignment from each biological dataset.** Axes are logarithmically scaled.
(EPS)

Table S1
**Simulation parameters and empirical statistics for the simulated datasets in our study.** The parameters used to evolve sequences on trees are listed for the fifteen 100-taxon models. The p-distance between two sequences is the normalized Hamming distance between the two sequences. “Setwise avg p-dist” is the average pairwise p-distance across all pairs of sequences in the true alignment, and “Setwise max p-dist” is the maximum pairwise p-distance across all pairs of sequences in the true alignment. “Gap” is the percentage of the true alignment matrix containing indels. “Cols” is the number of columns in the true alignment. “Avg gap len” is the average length of a gap, or contiguous string of indels, in the true alignment. The edgewise average (maximum) p-distance is the average (maximum) p-distance between the sequences labeling the endpoints of edges in the model tree. “Resolution” is the number of edges in the reference tree divided by 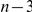, the maximum number of internal edges possible in any unrooted tree on 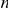 taxa.(PDF)

Table S2 **Empirical statistics for biological datasets.** Empirical statistics for the curated alignment are shown for all biological datasets. The curated alignment is used as the reference alignment. The columns from left to right show the dataset name, the number of taxa, the number of columns in the reference alignment, the average p-distance of the reference alignment, the maximum p-distance of the reference alignment, the percent indels of the reference alignment, the average gap length of the reference alignment, and the median gap length of the reference alignment. All biological datasets had a median gap length of 1.
(PDF)

Table S3
**Q-values from statistical tests comparing missing branch rates on 100-taxon model conditions.** We performed one-tailed paired t-tests with Benjamini-Hochberg correction [57] to see if ML(MAFFT)'s missing branch rate improved upon BeeTLe-Affine's. We also performed similar statistical tests to see if BeeTLe-Affine's missing branch rate improved upon MP(MAFFT)'s. 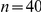 for each test.(PDF)

Table S4 **Normalized treelength scores obtained on the model tree and SATé-I tree.** For each of the three treelength criteria, we obtained treelength scores on either the model tree or SATé-I tree by constraining POY to solve a fixed-tree variant of the Generalized Sankoff Problem (see text for details). Treelength scores are normalized by the treelength score obtained by POY run under default settings. Averages (“Avg”) and standard errors (“SE”) are shown to either three or four decimal points. Model conditions are shown in the same order as in Figure 1.
(PDF)

Methods S1 **Supporting materials about methods.**
(PDF)
