# Supplementary material for: Treelength Optimization for Phylogeny Estimation
Source: PLoS One. 2012 Mar 19;7(3):e33104. doi: 10.1371/journal.pone.0033104 (PMC3307723; doi:10.1371/journal.pone.0033104)
Supplement: Table S1 — Simulation parameters and empirical statistics for the simulated datasets in our study. The parameters used to evolve sequences on trees are listed for the fifteen 100-taxon models. The p-distance between two sequences is the normalized Hamming distance between the two sequences. “Setwise avg p-dist” is the average pairwise p-distance across all pairs of sequences in the true alignment, and “Setwise max p-dist” is the maximum pairwise p-distance across all pairs of sequences in the true alignment. “Gap” is the percentage of the true alignment matrix containing indels. “Cols” is the number of columns in the true alignment. “Avg gap len” is the average length of a gap, or contiguous string of indels, in the true alignment. The edgewise average (maximum) p-distance is the average (maximum) p-distance between the sequences labeling the endpoints of edges in the model tree. “Resolution” is the number of edges in the reference tree divided by , the maximum number of internal edges possible in any unrooted tree on taxa. (PDF) [file pone.0033104.s003.pdf]

	Simulation Parameter			True Alignment Statistics (Set-wise)						PIMT Statistics (Branch-wise)		PIMT Statistics
**Model**	**Gap Len**	**Tree Height**	**Gap Prob**	**Avg. p-dist (%)**	**Max. p-dist (%)**	**Indels (%)**	**No. of Cols**	**Avg. Gap Len**	**Median Gap Len**	**Avg. p-dist (%)**	**Indels (%)**	**Resolution (%)**
100L1	long	30	2E-05	68.0	75.4	64.7	3044.7	12.9	9.4	29.0	1.2	99.7
100L2	long	20	4E-05	64.9	72.9	71.5	3843.2	14.2	10.2	24.5	1.6	99.7
100L3	long	20	2E-05	65.0	72.8	55.2	2347.2	11.1	8.2	24.4	0.8	99.6
100L4	long	15	3E-05	62.3	71.0	56.4	2459.7	11.2	8.1	20.5	0.8	99.7
100L5	long	2	2E-04	32.2	44.4	53.6	2281.9	11.4	8.5	4.3	0.7	95.9
100M1	medium	25	2E-05	66.4	74.1	44.0	1836.5	6.2	4.3	26.5	0.5	99.7
100M2	medium	20	4E-05	64.8	72.9	57.8	2486.7	7.4	5.0	24.4	0.9	99.6
100M3	medium	15	5E-05	62.6	71.0	55.1	2316.8	7.1	4.8	20.4	0.8	99.7
100M4	medium	7	1E-04	53.1	63.3	53.6	2262.9	7.0	4.8	12.7	0.8	99.3
100M5	medium	4	1E-04	45.0	56.4	38.9	1681.9	5.9	4.2	8.1	0.4	98.4
100S1	short	30	1E-05	67.6	74.9	16.1	1197.4	2.3	2.0	28.5	0.2	99.9
100S2	short	30	8E-06	67.9	74.9	14.7	1176.9	2.3	1.9	29.5	0.1	99.7
100S3	short	25	1E-05	66.6	74.0	14.2	1169.4	2.2	1.9	27.4	0.1	99.7
100S4	short	10	1E-04	58.0	67.5	40.4	1698.2	3.1	2.6	16.1	0.6	99.0
100S5	short	2	1E-03	32.0	43.6	57.2	2418.3	4.6	3.6	4.6	1.1	96.8
**max std dev**				2.0	1.5	5.9	400.2	1.3	1.3	2.6	0.2	2.0
