# Supplementary material for: Treelength Optimization for Phylogeny Estimation
Source: PLoS One. 2012 Mar 19;7(3):e33104. doi: 10.1371/journal.pone.0033104 (PMC3307723; doi:10.1371/journal.pone.0033104)
Supplement: Table S2 — Empirical statistics for biological datasets. Empirical statistics for the curated alignment are shown for all biological datasets. The curated alignment is used as the reference alignment. The columns from left to right show the dataset name, the number of taxa, the number of columns in the reference alignment, the average p-distance of the reference alignment, the maximum p-distance of the reference alignment, the percent indels of the reference alignment, the average gap length of the reference alignment, and the median gap length of the reference alignment. All biological datasets had a median gap length of 1. (PDF) [file pone.0033104.s004.pdf]

Dataset	No. of Taxa	No. of Cols	Avg. p-dist (%)	Max. p-dist (%)	Indels (%)	Avg. Gap Len
23S.M	278	10738	37.7	70.3	83.7	31.9
23S.M.aa_ag	263	10305	37.7	70.7	83.5	34.2
23S.E.aa_ag	144	8619	30.3	57.0	61.1	13.5
23S.E	117	9079	29.6	51.7	59.7	12.6
