# Supplementary material for: Treelength Optimization for Phylogeny Estimation
Source: PLoS One. 2012 Mar 19;7(3):e33104. doi: 10.1371/journal.pone.0033104 (PMC3307723; doi:10.1371/journal.pone.0033104)
Supplement: Table S3 — Q-values from statistical tests comparing missing branch rates on 100-taxon model conditions. We performed one-tailed paired t-tests with Benjamini-Hochberg correction [57] to see if ML(MAFFT)'s missing branch rate improved upon BeeTLe-Affine's. We also performed similar statistical tests to see if BeeTLe-Affine's missing branch rate improved upon MP(MAFFT)'s. for each test. (PDF) [file pone.0033104.s005.pdf]

Model	Affine	Simple-2	Simple-1
100L1	.0642	.0070	.0103
100L2	.1082	.0070	.0103
100M2	.0437	.0154	.0103
100S2	.1381	.0465	.0172
100M1	.1193	.0101	.0140
100S1	.1381	.0393	.0077
100L3	.1381	.0134	.0077
100S3	.1082	.0128	.0077
100M3	.0642	.0101	.0139
100L4	.1193	.0128	.0139
100S4	.0642	.0131	.0079
100M4	.0642	.0066	.0077
100S5	.0305	.0101	.0041
100M5	.0642	.0127	.0077
100L5	.1134	.0149	.0041
