# Supplementary material for: Treelength Optimization for Phylogeny Estimation
Source: PLoS One. 2012 Mar 19;7(3):e33104. doi: 10.1371/journal.pone.0033104 (PMC3307723; doi:10.1371/journal.pone.0033104)
Supplement: Table S4 — Normalized treelength scores obtained on the model tree and SATé-I tree. For each of the three treelength criteria, we obtained treelength scores on either the model tree or SATé-I tree by constraining POY to solve a fixed-tree variant of the Generalized Sankoff Problem (see text for details). Treelength scores are normalized by the treelength score obtained by POY run under default settings. Averages (“Avg”) and standard errors (“SE”) are shown to either three or four decimal points. Model conditions are shown in the same order as in Figure 1. (PDF) [file pone.0033104.s006.pdf]

Model	ML(MAFFT) better than BeeTLe-Affine	BeeTLe-Affine better than MP(MAFFT)
100L1	.1137	.0021
100L2	.0003	.0038
100M2	$<$.0001	.0092
100S2	$<$.0001	.3506
100M1	$<$.0001	.0184
100S1	$<$.0001	.6673
100L3	$<$.0001	.4495
100S3	.0002	.0433
100M3	.0001	.1493
100L4	$<$.0001	.7245
100S4	.0011	.0021
100M4	.0034	.0077
100S5	.1344	.1264
100M5	.0013	.0855
100L5	.0032	.5433
