# Supplementary material for: Treelength Optimization for Phylogeny Estimation
Source: PLoS One. 2012 Mar 19;7(3):e33104. doi: 10.1371/journal.pone.0033104 (PMC3307723; doi:10.1371/journal.pone.0033104)
Supplement: Methods S1 — Supporting materials about methods. (PDF) [file pone.0033104.s007.pdf]

Supporting Information: Treelength Optimization for Phylogeny Estimation

Kevin Liu[1] and Tandy Warnow[1,*]

1 Department of Computer Science, University of Texas at Austin, Austin, TX, USA

∗ E-mail: tandy@cs.utexas.edu

Supporting Materials and Methods

To produce alignments for our two-phase analyses, we used ClustalW version 2.0.4, MAFFT version 6.240, Muscle version 3.7, Prank+GT using Prank version 080904 and using RAxML(MAFFT) as a guide tree, ProbconsRNA version 1.1 with a UPGMA guide tree, resulting in a Probtree alignment (as described in [1]), and Opal version 1.0.2, which required Java version 1.6.0_02.

- ClustalW:
  ```
  clustalw2 -align -infile=<input>
  -outfile=<output> -output=fasta
  ```
- MAFFT
  ```
  mafft --localpair --maxiterate 1000
  --quiet <input> > <output>
  ```
- Muscle:
  ```
  muscle -in <input> -out <output> -quiet
  ```
- Prank+GT:
  ```
  prank -d=<input> -o=<output>
  -t=<RAxML(MAFFT) guide tree> -noxml -notree
  -nopost +F -matinitsize=5 -uselogs
  ```
- Probtree:
  ```
  probcons -g <input guide tree>
  <input sequences> > <output>
  ```

- Opal:
  `java -jar Opal.1.0.2.jar --in <input>`
  `--out <output>`

To compute the UPGMA guide tree for Probtree, we used the procedure of Nelesen et al. [1]. First, we used custom software (available upon request) to compute a pairwise distance matrix. Each entry in the matrix corresponds to the optimal cost of a Needleman-Wunsch alignment [2] between two unaligned sequences, where each substitution has a cost of 1 and a gap of length k is penalized by cost k. Then, we used the following script with PAUP* version 4.0b10 to compute a midpoint-rooted UPGMA tree from the distance matrix.

```
#NEXUS
begin taxa;
  dimensions ntax=<number of taxa>;
  taxlabels <space-delimited list of taxon labels>;
end;
begin distances;
  format triangle=upper
         nodiagonal;
matrix
<upper triangular pairwise distance matrix,
 where entries are space-delimited and
 each row begins with a taxon label>
;
end;
begin paup;
  set root=midpoint;
  upgma showtree=no brlens=no treefile=<output> replace=yes;
end;
```

MP trees were produced using a custom script (implemented by Olaf Bininda-Emonds and modified by Tiffani Williams; available at `www.cs.utexas.edu/~phylo/software/ratchet.pl`) in combination with PAUP* version 4.0b10 to perform an MP Ratchet

analysis of a fixed alignment. Each Ratchet analysis performed TBR operations for 100 iterations with 25% character re-weighting. We then computed the majority consensus of most parsimonious trees from each Ratchet analysis. ML trees were produced on the alignments using RAxML version 7.2.6 with the following command.

- RAxML:
  ```
  raxmlHPC -m GTRMIX -w <work dir>
  -n <identifying suffix> -s <input>
  ```

All POY-related analyses were performed using POY version 4.1.2. We ran POY as a heuristic for the Generalized Sankoff Problem using the following script:

```
read ("<sequences data
        file in FASTA format>")
transform (tcm:"<matrix file
                 with substitution,
                 gap extend
                 penalties>",
           gap_opening:
             <gap opening cost>)
(* replace above with
   transform (tcm:(1,1),
              gap_opening:0)
   for Simple-1 penalty *)
build (1)
swap ()
select ()
report (trees:(_cost),
        asciitrees, ia)
report ("poy.trees", trees)
report ("poy.treestats", treestats)
report ("poy.data", data)
report ("poy.diagnosis", diagnosis)
report ("poy.implied_alignments",
```

```
        implied_alignments)
exit ()
```

We also used POY to compute a treelength score on a fixed topology. This analysis was performed with the following script:

```
read ("<sequences data file
        in FASTA format>")
read ("<binary tree to score
        in PAREN format>")
transform (tcm:"<matrix file with
                 substitution, gap
                 extend penalties>",
           gap_opening:
                    <gap opening cost>)
(* replace above with
   transform (tcm:(1,1), gap_opening:0)
   for Simple-1 penalty *)
rediagnose()
select ()
report ("POY_SCORE.trees", trees)
report ("POY_SCORE.treestats", treestats)
report ("POY_SCORE.data", data)
report ("POY_SCORE.diagnosis", diagnosis)
report ("POY_SCORE.implied_alignments",
         implied_alignments)
exit ()
```

To run SATé, we used the SATé version 1.1 distribution provided as part of the online supplementary materials of Liu et al.'s prior study [3]. The following command was used to run SATé.

- SATé:
  ```
  ./sate_basic.pl -r <name of run>
  -w <empty temporary work directory with full path>
  ```

```
-d <input unaligned sequences file with full path>
-l 1 -s 1 -a 5
```

User-friendly software to perform SATé-II analyses can be obtained from `phylo.bio.ku.edu/software/sate/sate.html`. Instructions on how to run this software are included with the latest distribution.

We used custom software provided by Morgan Price to compute the missing branch rate between two trees (available at `www.microbesonline.org/fasttree/treecmp.html`). The software used to compute alignment SP-FN error between two alignments is available as part of the online supplementary material of Liu et al.'s prior study [3].

References

1. Nelesen S, Liu K, Zhao D, Linder CR, Warnow T (2008) The effect of the guide tree on multiple sequence alignments and subsequent phylogenetic analyses. In: Pacific Symposium on Biocomputing. volume 13, pp. 15-24.

2. Needleman SB, Wunsch CD (1970) A general method applicable to the search for similarities in the amino acid sequence of two proteins. J Mol Biol 48: 443–453.

3. Liu K, Linder CR, Warnow T (2010) Multiple sequence alignment: a major challenge to large-scale phylogenetics. PLoS Currents: Tree of Life 2:RRN1198.
